# Clinical characteristics of patients from Quebec, Canada, with Morquio A syndrome: a longitudinal observational study

**DOI:** 10.1186/s13023-020-01545-y

**Published:** 2020-09-29

**Authors:** Lina Moisan, David Iannuzzi, Bruno Maranda, Philippe M. Campeau, John J. Mitchell

**Affiliations:** 1 Division of Medical Genetics, Montreal Children’s Hospital, McGill University Health Centre, Montreal, QC Canada; 2 Medical Genetics Division, Centre Hospitalier Universitaire Sherbrooke, Sherbrooke, QC Canada; 3 Medical Genetics Division, Department of Pediatrics, Centre Hospitalier Universitaire Sainte-Justine, Montreal, QC Canada; 4 Division of Pediatric Endocrinology, Montreal Children’s Hospital, McGill University Health Centre, A04.6309, 1001 Decarie, Montreal, QC Canada

**Keywords:** Canada, Founder effects, Genetic disorders, Mucopolysaccharidosis IVA, Activities of daily living, Walk test

## Abstract

**Background:**

Morquio A syndrome is a rare, autosomal recessive, progressively debilitating disorder, with multi-system impairments and high medical burden. Quebec, Canada has a large Morquio A population, which is considered unique due to the presence of founder pathogenic variants. The objectives of this study were to document the genetic and clinical heterogeneity of patients with Morquio A in Quebec, to better characterize the phenotype of those with the French Canadian founder pathogenic variant (NM_000512.5: c.1171A>G, p.Met391Val), and to describe the natural history of the patients treated with elosulfase alfa enzyme replacement therapy. Patients with Morquio A were genotyped for pathogenic variants in the lysosomal enzyme *N*-acetylgalactosamine-6-sulfatase. Clinical data were retrospectively collected from medical charts of patients and included medical history, height, physical examination, respiratory function tests, electrocardiogram, echocardiogram, endurance in the 6-min walk test (6MWT), and activities of daily living (ADL) as assessed by the Mucopolysaccharidosis Health Assessment Questionnaire (MPS-HAQ). Longitudinal data were collected retrospectively and prospectively for patients treated with elosulfase alfa.

**Results:**

A total of 33 patients, aged 5–63 years, were included in the analysis. Patients with the founder pathogenic variant (n = 17) generally exhibited a non-classical form of Morquio A. As compared with patients with a non-founder pathogenic variant (n = 16), these patients were generally taller, had greater endurance and were better able to perform ADL. However, they still had significant musculoskeletal disease. Most of the 26 patients treated with elosulfase alfa, regardless of pathogenic variant, showed improvements in endurance and ADL. After 5 to 12 months of treatment, the mean improvement from baseline in the 6MWT was 23% and 10 of 14 patients improved in at least one MPS-HAQ domain. Endurance and ADL generally continued to improve or maintained stable in the long term (up to 7 years). Four out of 19 treated patients with echocardiogram data at follow-up showed progression of cardiac disease.

**Conclusions:**

In Quebec, Canada, Morquio A frequently manifests as a non-classical form of the syndrome due to a founder effect. Patients treated with elosulfase alfa generally show long-term improvement or stability in endurance and function, regardless of pathogenic variant.

## Background

Morquio A syndrome, also called mucopolysaccharidosis IVA (MPS IVA), is an ultra-rare, genetically transmitted lysosomal storage disorder caused by a deficiency of the *N*-acetylgalactosamine-6-sulfatase (GALNS) enzyme [1, 2]. Deficiency of the GALNS enzyme manifests as a failure to degrade glycosaminoglycans (GAGs), namely keratan sulfate (KS) and chondroitin-6-sulfate (C6S), resulting in excessive accumulation of these GAGs [2] and subsequent activation of secondary pathways of inflammation. The result of the GAG accumulation is a progressively debilitating and potentially life-threatening disorder, with multi-system impairments and a high medical burden [3]. The main organ affected is bone, with accumulation of KS and C6S resulting in impaired development of cartilage and bone and eventual systemic skeletal spondyloepiphyseal dysplasia [2, 4, 5]. The clinical presentation is heterogeneous [6] and typically includes marked skeletal and joint abnormalities, coarsening of facial features, short-trunk dwarfism with short neck, restrictive and obstructive lung disease, impaired cardiac function, hearing and vision loss, abdominal manifestations (hepatomegaly, hernias), waddling gait leading to increased fall risk, and dental abnormalities [4, 5, 7]. Classical forms of Morquio A syndrome are also associated with fatigue, sleep apnea, shortness of breath, tracheal obstruction, and recurrent upper respiratory tract infections [5]. Over time, the progressive loss of functional capacity and endurance leads to reduced quality of life [3]. Many individuals become wheelchair-dependent and require multiple surgeries [3, 6]. Morquio A patients typically have a shortened life expectancy, with the main cause of death being respiratory failure [6, 8].

Available prevalence data for Morquio A syndrome are derived from epidemiologic studies conducted in individual countries. The birth prevalence ranges from 1 per 71,000 in the United Arab Emirates to 1 in 500,000 in Japan, with most countries having < 0.5 cases per 100,000 live births [9]. Morquio A appears to be more prevalent than in most countries in Quebec, Canada, although precise numbers are not available. In 1973, a survey of the main hospitals in Quebec conducted by Gadbois et al. identified 48 patients with the disease distributed within 27 families [10]. The high prevalence of Morquio A in Quebec is explained by the fact that French Canadians represent a unique population with a higher rate of some recessive genetic diseases that reflects the presence of founder pathogenic variants [11].

Treatment of Morquio A syndrome has traditionally been supportive in nature, with the goals of managing symptoms with physical therapy and surgery [7]. Enzyme replacement therapy with elosulfase alfa (VIMIZIM^®^, BioMarin Pharmaceutical Inc, CA, USA) was approved by the US Food and Drug Administration in February 2014, by the European Medicines Agency in April 2014, and by Health Canada in September 2014 [12]. Approvals were based on the findings of a 24-week, placebo-controlled, Phase 3 trial that randomized 176 patients [13]. Once-weekly intravenous infusions of elosulfase alfa (2 mg/kg) significantly improved endurance based on the 6-min walk test (6MWT), and the most common adverse events were infusion-related reactions and hypersensitivity [13]. These improvements were sustained over 120 weeks among patients participating in an extension study of the trial [14]. Another long-term study showed no trends towards decreasing endurance and maintained tolerability and safety over 5 years in 17 patients, many of whom elected to be treated with home infusions [15].

Canadian patients with Morquio A syndrome face substantial barriers to accessing elosulfase alfa treatment because of the absence of an orphan drug plan in Canada and marked differences in approval processes across provinces [16]. Given the cost of orphan medications and possible pharmacogenetic effects in the Quebec population, data supporting clinical efficacy are critical. This report documents the genetic and clinical heterogeneity of Morquio A syndrome in a series of patients from Quebec, and describes longitudinal data from those treated with elosulfase alfa.

## Methods

### Study design and objectives

This was a longitudinal observational study of patients with Morquio A syndrome in Quebec, Canada. All patients with a confirmed Morquio A diagnosis who provided consent to participate in the study are included.

The main objectives of the study were to document the genetic and clinical heterogeneity of the Morquio A patient population in Quebec in general, to characterize the phenotype of patients with the French Canadian founder pathogenic variant (NM_000512.5: c.1171A>G, p.Met391Val), and to describe the disease course of those treated with elosulfase alfa.

The study protocol was approved by the institutional review board at McGill University. All patients (or their guardians) provided written informed consent before study entry. The study was conducted in accordance with the Declaration of Helsinki and International Conference of Harmonization Good Clinical Practice guidelines [17].

### Genetic testing

All patients were genotyped for pathogenic variants in *GALNS*. Patients were stratified by the presence or absence of the French Canadian founder pathogenic variant c.1171A>G, p.Met391Val. Patients with at least one copy of this pathogenic variant were included in the founder pathogenic variant group, regardless of the presence of other pathogenic variants (because of suggestions that, in compound heterozygotes, the phenotype of the allele with residual activity will be displayed [18]).

### Data collection

Clinical data were retrospectively collected from November 2009 to December 2014 and prospectively from January 2015 to May 2019 via chart review of medical records. Clinical data were collected approximately every 6 months or at every clinic visit [7]. Clinical assessments for which data are presented include medical history, height (z-score was calculated [19]), physical examination, respiratory function tests (forced expiratory volume in 1 s [FEV_1_] and forced vital capacity [FVC]), electrocardiogram, echocardiogram, endurance, and activities of daily living (ADL). Endurance was assessed by the 6MWT [7]. ADL were assessed by the MPS Health Assessment Questionnaire (MPS-HAQ; originally developed for patients with MPS I [13]). The MPS-HAQ assesses self-care (eating/drinking, dressing, bathing, grooming, tooth brushing, and toileting), mobility (dexterity, mobility, walking, stair climbing, and gross motor skills), and caregiver-assistance required (Care Services) [20]. Total self-care and mobility domain scores range from 0 (not difficult at all) to 10 (extremely difficult) and 11 (unable to do). The total Care Services domain score ranges from 13 (independent) to 52 (complete assistance required) [20]. Decreasing scores imply improvements.

For some of the patients, some data could retrospectively be collected from the MOR-001 natural history study (NCT00787995; [1, 21]), the Phase 3 MOR-004 and MOR-005 studies (NCT01275066 and NCT01415427; [13, 14]), and the Phase 2 MOR-008 study (NCT01609062; [22]), and were included in this study.

### Statistical analysis

Descriptive statistics were used to summarize data. Baseline data are presented for all patients. For patients initiating elosulfase alfa therapy, data at baseline and at last follow-up are presented. Baseline was defined as the closest measurement prior to, or on the day of, first treatment for patients treated with elosulfase alfa, and as the first measurement recorded for the other patients. For baseline measurements and change from baseline, the founder pathogenic variant group was compared with the non-founder pathogenic variant group by an unpaired t test of unequal variance (*P* < 0.05 was considered significant). Missing data was not included in the analysis.

## Results

### Demographic and baseline clinical characteristics

We evaluated 33 patients with Morquio A syndrome from Quebec who provided consent to participate in the study (Table 1). There were more males (58%) than females (42%) in the overall population. Mean age was 25.9 years (range: 5–63 years; median: 21 years), and the mean height of adults (n = 23) was 1.23 m. As determined based on height data, compared with Morquio A-specific growth charts [23], phenotype was determined as classical in 12 patients, non-classical in 16 patients, and intermediate in five patients (Table 1). Four patients previously participated in the MOR-001 natural history study (patients 2, 19, 26, and 27), four in the MOR-004 and MOR-005 studies (patients 3, 7, 25, and 29), and three in the MOR-008 study (patients 26, 27, and 34). Twenty six patients had received treatment with elosulfase alfa. In these patients, elosulfase alfa was administered according to labeling as 2 mg/kg intravenously once every week.

**Table 1:** Baseline characteristics of individual patients with Morquio A syndrome

ID	*GALNS* pathogenic variants		Sex	Age (years)	Height z-score^b^	Adult height (m)^a^	Elosulfase alfa treatment	Phenotype^c^
Founder pathogenic variant group								
1	1171A>G	121A>T	M	43	− 4.1	1.47	No	NC
3	1171A>G	901G>T	F	23	− 5.8	1.23	Yes	NC
7	**1171A**>**G**	**1171A**>**G**	F	25	− 4.8	1.25	Yes	NC
17	1171A>G	405_422+1del	F	60	− 6.8	1.19	No	NC
18	1171A>G	841_867_del	F	36	− 4.3	1.36	Yes	NC
20	**1171A**>**G**	**1171A**>**G**	F	14	− 2.5	–	Yes	NC
21	1171A>G	405_422 + 1del	F	58	− 6.5	1.25	Yes	NC
22	1171A>G	121A>T	M	7	− 7.8	–	Yes	I
24	1171A>G	121A>T	M	45	− 6.1	1.32	Yes	I
26	1171A>G	1354T>A	M	21	− 1.6	1.67	Yes	NC
27	1171A>G	1354T>A	F	17	− 1.6	–	Yes	NC
29	1171A>G	404_422del19	F	17	− 5.4	–	Yes	NC
30	1171A>G	1157G>A	M	36	− 6.1	1.32	Yes	I
31	**1171A**>**G**	**1171A**>**G**	M	40	− 4.9	1.39	Yes	NC
32	1171A>G	121A>T	F	40	− 5.5	1.27	No	NC
33	**1171A**>**G**	**1171A**>**G**	M	23	− 3.1	1.41	No	NC
34	**1171A**>**G**	**1171A**>**G**	F	16	− 1.4	–	Yes	NC
Non-founder pathogenic variant group								
2	244+1G>T	901G>T	M	27	− 7.5	1.21	Yes	C
4	901G>T	121A>T	M	12	− 7.6	–	Yes	C
5	901G>T	121A>T	M	10	− 4.6	–	Yes	I
6	901G>T	704C>A	F	9	− 7.1	–	Yes	C
8	901G>T	319G>A	M	16	− 6.7	–	Yes	C
9	121A>T	841_867_del	F	63	− 6.9	1.05	Yes	I
11	901G>T	405_422+1_del	M	27	− 11.1	0.92	Yes	C
12	901G>T	405_422+1_del	M	25	− 10.4	0.98	Yes	C
13	901G>T	121A>T	F	35	− 8.0	1.15	Yes	NC
14	1157G>A	1157G>A	M	15	− 5.6	–	No	C
15	1157G>A	1157G>A	M	21	− 11.0	0.92	No	C
16	1157G>A	1157G>A	M	19	− 11.5	–	No	C
19	244T>C	244T>C	F	20	− 1.9	1.36	Yes	NC
23	901G>T	901G>T	M	5	− 5.2	–	Yes	C
25	406_424del19	1480A>G	M	15	− 7.2	–	Yes	C
28	938C>T	938TC>T	M	16	− 7.8	–	Yes	C

Bold indicates a homozygote founder pathogenic variant

Patient 10 withdrew consent and is therefore not included in the analysis

1171A>G: founder pathogenic variant; F: female; *GALNS*: *N*-acetylgalactosamine-6-sulfatase gene; ID: patient identification number; M: male; NA: not available

^a^Adult height is only presented for patients ≥ 18 years of age

^b^Z-score was calculated at https://apps.cpeg-gcep.net/quickZ_WHO

^c^Phenotypes were determined based on Morquio A-specific growth charts [23]: patients were classified as classical (C) when height was ≤ 50th % ile, as intermediate (I) when height was > 50th and < 75th %ile, and as non-classical (NC) when height was ≥ 75th %ile

Ejection fraction was available for 15 patients, and was within normal limits (55–70%) in these patients. Lung function data were available for 20 patients; FEV_1_/FVC ratio was within the normal range (> 70%) in most of these patients (Table 2). It should be noted that for many patients, it was difficult to interpret FEV_1_ and FVC data as % predicted cannot be calculated for individuals with short stature for whom norms are not available. Eleven out of 26 patients with available echocardiogram data had evidence of cardiac abnormalities. These included ten patients with valve disease and one patient with ascending aorta dilatation that worsened over time (see footnote Table 2 for details). These abnormalities were of clinical significance in six patients (patients 2, 8, 11, 19, 25, and 31). Two patients (11 and 12), both with a non-founder pathogenic variant and significant respiratory involvement died during the study due to cardiac arrest. These patients were siblings and died 1 year apart. They both had classical disease and significant respiratory involvement. Patient 11 had a previous cardiac arrest, and death occurred during activity, likely due to arrhythmia. Patient 12 died in same manner. The exact cause of death could not be determined, as there was no autopsy. Four patients (see footnote Table 2 for details) had difficulty sustaining respiratory efforts and pulmonary function could not be measured.

**Table 2:** Cardiac and lung function of individual patients at baseline and after elosulfase alfa treatment

ID	Cardiac abnormalities at baseline^e^	Ejection fraction (%)^a^			Lung function^b^ FEV_1_/FVC (%)^c^		
		Baseline	Elosulfase alfa treatment		Baseline	Elosulfase alfa treatment	
			Follow-up	Duration		Follow-up	Duration
Founder pathogenic variant group							
1	No	70	NT		73	NT	
3	No	NA	65	6.5 years	91	91	2 years
7	No	NA	65	4.5 years	85	85	6 years
17	NA	NA	NT		NA	NT	
18	No	>50	60	3 years	88	86	3 years
20	Yes	NA	67	4 years	81	88	7 years
21	Yes	75	NA		84	NA	
22	NA	NA	NA		87	92	2.5 years
24	No	NA	70	6 months	NA	80	2 years
26	No	NA	61	2 years	86	82	6 years
27	No	NA	65	5 years	83	83	5 years
29	No	59	49	5 years	91	91	6.5 years
30	No	60	NA		NA		
31	Yes^d^	NA	70	6 months	NA	86	2 years
32	No	74	NT		NA	NT	
33	No	NA	NT		86	NT	
34	NA	NA	60	1 year	89	89	6 years
Non-founder pathogenic variant group							
2	Yes	NA	NA		91	90	1.5 years
4	NA	NA	NA		NA	NA	
5	No	NA	59	1 year	NA	NA	
6	Yes	67	NA		NA	NA	
8	Yes	78	82	3 years	84	100	3 years
9	Yes	70	65	3 years	79	77	3 years
11	Yes	70	65	1 year	NA	NA	
12	No	55	55	3 years	NA	68	3 years
13	No	NA	NA		NA	NA	
14	NA	60	NT		71	NT	
15	NA	78	NT		101	NT	
16	NA	69	NT		71	NT	
19	Yes	NA	NA		85	86	4 years
23	No	NA	NA		NA	NA	
25	Yes	62	NA		98	NA	
28	Yes	NA	79	1 year	NA	92	1 year

For patients who received elosulfase alfa, the baseline measurement (closest measurement prior to, or on the day of, first treatment) was included. For other patients, the first measurement recorded is included in the table

*FEV*_*1*_ forced expiratory volume in 1 s, *FVC* forced vital capacity, *ID* patient identification number, *NA* not available, *NT* no elosulfase alfa treatment

^a^Normal range 55–70%

^b^Pulmonary abnormalities included the following: Patients 8, 9, 25, and 29 had difficulty sustaining respiratory efforts

^c^Normal range ≥ 70%

^d^Baseline echographic examination of subject 31 was performed 4 months after initiating elosulfase alfa

^e^Cardiac abnormalities included the following: Patient 2 had a mitral valve insufficiency and aortic valve stenosis; Patient 6 had a thickened aortic valve and mitral valve with trivial mitral regurgitation; Patient 8 had ascending aorta dilatation that worsened overtime; Patient 9 had atrioventricular valve sclerosis; Patient 11 had mitral valve thickening and aortic valve replacement; Patient 19 had pulmonary valve stenosis and pulmonary artery dilation; Patient 20 had mild pulmonary and tricuspid regurgitation; Patient 21 had atrioventricular valve sclerosis; Patient 25 had moderate mitral valve regurgitation and left ventricular outflow obstruction; Patient 28 had a slight reformation of the mitral valve; and Patient 31 had a bicuspid aortic valve and aortic root dilation

Genetic data were available for all patients; 17 patients expressed at least one copy of the French Canadian founder pathogenic variant (c.1171A>G, p.Met391Val), five of which were homozygotes, and 16 patients had only non-founder pathogenic variants (Table 1).

### Disease traits related to founder pathogenic variant

**Fig. 1:**
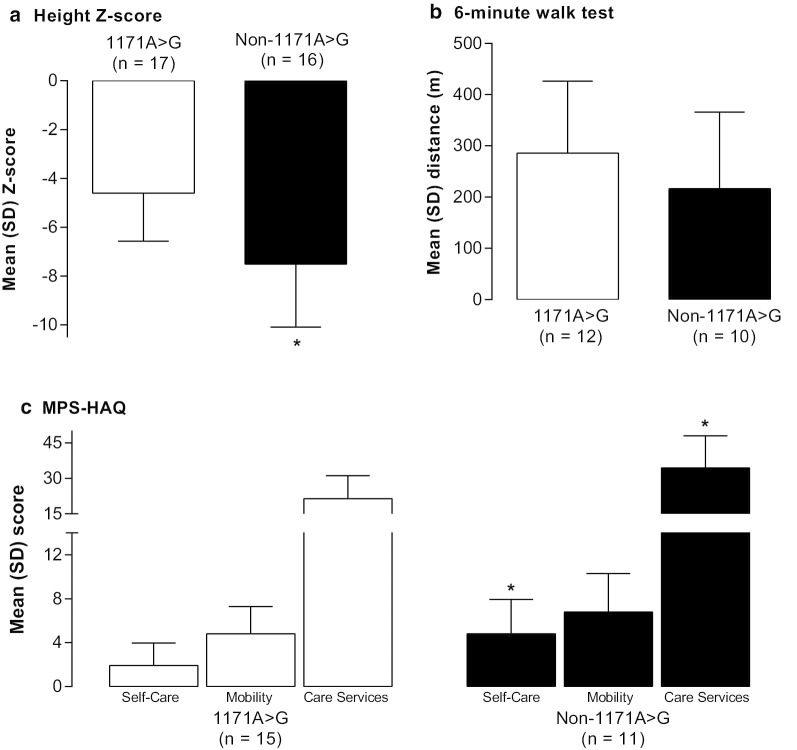
Baseline measures. **a** Mean ± SD height z-score (for children) or SD from the average of people of the same age and sex (for adults), **b** Mean ± SD distance in the 6-min walk test, and **c** mean ± SD score for the self-care, mobility, and care services domains of the MPS-HAQ. 1171A>G: founder pathogenic variant; MPS-HAQ: Mucopolysaccharidosis Health Assessment Questionnaire; SD: standard deviation. *P = 0.001

Patients with the founder pathogenic variant tended to exhibit a non-classical form of Morquio A syndrome, while most patients without the founder pathogenic variant had a classical phenotype (Tables 1, 2 and 3, Fig. 1). Although most patients, regardless of pathogenic variant, were substantially shorter than normalized World Health Organization values, patients with the founder pathogenic variant were significantly (*P* = 0.001) taller by z-score than most patients in the non-founder group (Fig. 1a). Nevertheless, many of the founder patients still had significant musculoskeletal disease. Patients with the founder pathogenic variant could also walk slightly farther during the 6MWT than non-founder patients, though the difference was not statistically significant (*P* = 0.281) (Fig. 1b). In addition, patients with the founder pathogenic variant had better ability to perform ADL, as measured with the MPS-HAQ, compared with patients with a non-founder pathogenic variant; the domains of self-care, mobility, and care services were all better (lower scores) for those with the founder pathogenic variant (*P* = 0.001, *P* = 0.075, and *P* = 0.001, respectively) (Fig. 1c). Finally, patients with the founder pathogenic variant showed less cardiac abnormalities and impairments in respiratory function than those with non-founder pathogenic variants.

**Table 3:** Baseline 6MWT and MPS-HAQ results of individual patients

ID	Endurance	MPS-HAQ		
	6MWT (m)	SC	MOB	CS
Founder pathogenic variant group				
1	27	3.1	6.6	22
3	321	0.2	4.7	23
7	138	0.7	6.3	26
17	NM	2.4	5.7	14
18	371	NA	NA	NA
20	246	1.9	3.3	23
21	330	1.2	2.2	23
22	NA	NA	NA	NA
24	230	1.2	5.7	13
26	423	0.4	2.1	15
27	338	0.04	1.6	14
29	123	2.1	4.5	21
30	NM	5.2	8.6	20
31	535	1.5	2.5	13
32	NA	0.8	2.7	13
33	NM	0.3	5.6	33
34	347	NA	NA	NA
Non-founder pathogenic variant group				
2	NM	8.8	9.8	47
4	289	2.7	1.2	17
5	458	1.5	0.9	19
6	261	NA	NA	NA
8	225	NA	NA	NA
9	NM	9.5	10.0	51
11	NM	9.2	10.0	52
12	NM	2.9	8.3	42
13	439	0.4	3.1	13
14	141	4.4	8.0	37
15	NM	7.6	10.0	48
16	25	5.2	9.0	37
19	171	3.2	5.6	36
23	NA	4.1	1.5	30
25	120	5.1	8.7	28
28	36	NA	NA	NA

For patients who received elosulfase alfa, the baseline measurement (closest measurement prior to, or on the day of, first treatment) was included. For other patients, the first measurement recorded is included in the table

*6MWT* 6-min walk test, *CS* care services, *ID* patient identification number, *MOB* mobility, *MPS-HAQ* Mucopolysaccharidosis Health Assessment Questionnaire, *NA* not available, *NM* not mobile, *SC* self-care

### Longitudinal data of patients treated with elosulfase alfa

Of the 26 patients who had received treatment with elosulfase alfa; 13 had a founder pathogenic variant and 13 had a non-founder pathogenic variant. The duration of treatment varied from 6 months to 7 years.

**Fig. 2.**
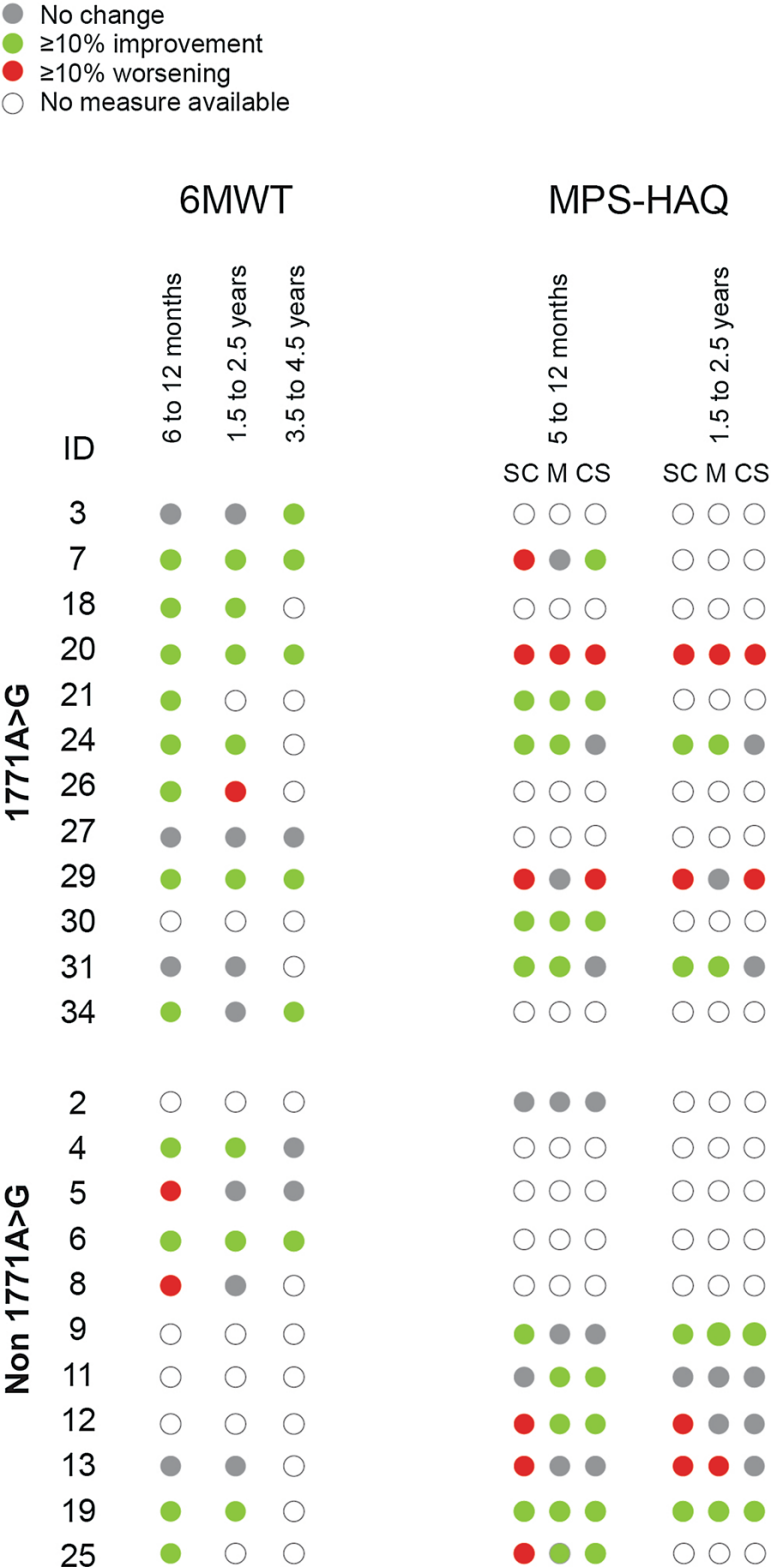
6MWT and MPS-HAQ results in patients treated with elosulfase alfa after 5 or 6 to 12 months, 1.5 to 2.5 years, and (for 6MWT) 3.5 to 4.5 years. Individual patient responses in 6MWT and MPS-HAQ were classified as no change from baseline, ≥ 10% improvement (longer distance on the 6MWT or lower score on the MPS-HAQ) from baseline, ≥ 10% worsening (shorter distance on the 6MWT or higher score on the HAQ) from baseline, or no measure available. Note that the closest measurement to, but not exceeding, the largest time of treatment was included. For 6MWT, some patients (5, 13, and 31) had normal 6MWT for height [29], and patient 26 had a decrease in 6MWT at 1.5 to 2.5 years because of back surgery (but returned to normal 6MWT for height in later years). 1171A>G: founder pathogenic variant; 6MWT: 6-min walk test; CS: care services; MPS-HAQ: Mucopolysaccharidosis Health Assessment Questionnaire; M: mobility; SC: self-care

For patients treated with elosulfase alfa and assessed between 5 and 12 months after the start of treatment, changes from baseline in 6MWT and MPS-HAQ results were variable (Fig. 2). However, there was no significant difference between patients with the founder or non-founder pathogenic variant at 5 to 12 months for either 6MWT (*P* = 0.896) or MPS-HAQ (self-care *P* = 0.1134; mobility *P* = 0.3423; care services *P* = 0.806). Importantly, many patients from both groups showed improvements in the 6MWT within this timeframe (Fig. 2). The mean improvement in the 6MWT for all patients, regardless of pathogenic variant, was 23%. For the MPS-HAQ, we observed ≥ 10% improvement (decrease in score) in at least one domain in 10 of 14 patients with available data, regardless of pathogenic variant; seven patients improved in at least two domains, and three patients improved in all three domains. Some patients had low baseline scores with little room for improvement. In general, patients showing initial improvements were able to sustain these over long-term treatment (Fig. 2).

**Fig. 3:**
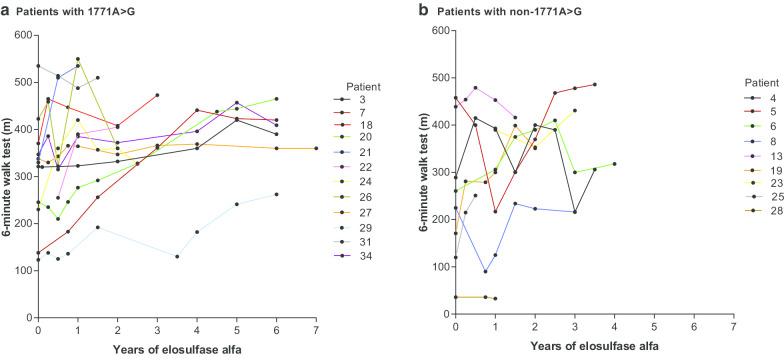
Individual patient 6-min walk test distances over time following treatment with elosulfase alfa. Note for patient 28, distance improved to 153 m, but this was with a walker and has not been included on the figure. 1171A>G: founder pathogenic variant

**Fig. 4:**
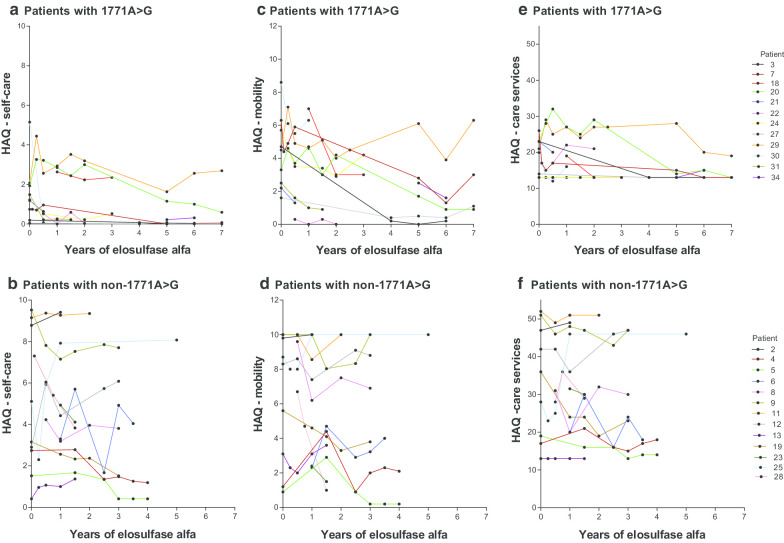
Individual patient MPS-HAQ scores over time for the domains of self-care, mobility, and care services, following treatment with elosulfase alfa. Note that lower scores indicate a better outcome. 1171A>G: founder pathogenic variant; HAQ: Health Assessment Questionnaire

Patients treated with elosulfase alfa for a longer time showed improvements in the 6MWT regardless of pathogenic variant (Fig. 3a, b). In both groups, improvements in endurance were generally maintained, or continued to improve, over many years of treatment. MPS-HAQ results in the long term were more variable, but several patients showed long-term improvements, regardless of pathogenic variant (Fig. 4). Overall, the spread of MPS-HAQ scores for patients with non-founder pathogenic variants was greater than for those with the founder pathogenic variant (Fig. 4), perhaps because phenotypes within the founder group were more uniform than within the non-founder group, which showed a wider variation of phenotypes.

Overall, ejection fraction and FEV_1_/FVC ratio of patients treated with elosulfase alfa remained stable over time (for up to 7 years), regardless of pathogenic variant (Table 2). Four out of 19 treated patients with echocardiogram data at follow-up showed progression of cardiac disease (patients 2, 11, 19, and 25, all from the non-founder pathogenic variant group).

Longitudinal data of untreated patients are not presented as data for these patients were too limited to make any meaningful comparison with treated patients.

## Discussion

This is the first report of Morquio A syndrome in French Canadians from Quebec in over 45 years. In 1973, Gadbois and colleagues described the physical appearance and biochemical characteristics of 48 Quebecois with the disease [10]. Our observations expand on these data and highlight the phenotypic variability in this unique population. Over half of our patient sample expressed the French Canadian founder pathogenic variant in *GALNS* (i.e., c.1171A>G, p.Met391Val). This pathogenic variant appears to be associated with a more non-classical form of Morquio A syndrome than most non-founder pathogenic variants, as shown by a greater mean adult height, a better endurance and ability to perform ADL, and less cardiac abnormalities and impairments in lung function. The majority of patients received treatment with elosulfase alfa. These patients generally showed improvements over time in 6MWT and MPS-HAQ outcomes, regardless of whether or not they had the French Canadian founder pathogenic variant.

The improvements in the 6MWT and MPS-HAQ observed in the patients receiving elosulfase alfa were similar to those observed in international clinical trials. In the Phase 3 trial, patients randomized to elosulfase alfa 2 mg/kg for 24 weeks (n = 58) showed a mean improvement in 6MWT distance of 17.9% over baseline (versus 6.4% for placebo; n = 59) [13]. This is similar to the findings in our study (23% improvement over baseline after 5–12 months, regardless of pathogenic variant), and above the mean minimal clinically important difference in the 6MWT estimated for respiratory, cardiovascular, and muscular diseases (7–9%) [4]. In the Phase 3 extension trial, 6MWT remained stable with elosulfase alfa treatment over 120 weeks [14], while two sequential open-label studies in 17 patients showed sustained improvements over up to 5 years of treatment [15]. These clinical trial results are also similar to our findings of 6MWT improvements or maintenance over a period of up to 7 years. In addition, we observed improvements in MPS-HAQ outcomes, similar to those seen in the Phase 3 clinical trial [20, 24]. It should be kept in mind that 6MWT distance increases with age and height in children, which may partly explain long-term increases in the test in younger patients [25, 26]. Nevertheless, long-term increases or stability in the 6MWT were also seen in most adult patients. These long-term results should be interpreted in light of the progressive nature of Morquio A. Published natural history data have shown a gradual decline in 6MWT and MPS-HAQ results over time in the absence of treatment [1, 14, 20]. The finding that most patients in our study showed stable or improved endurance and/or MPS-HAQ scores over time indicates that elosulfase alfa can slow down the gradual regression in these measures.

The findings of our study contribute to a better understanding of the clinical characteristics and genetic underpinnings in Quebecois, as well as the natural history of the disease in those receiving elosulfase alfa. Although elosulfase alfa, the first and only medical therapy available to modify the disease at a cellular level, has been approved in Canada for patients with Morquio A syndrome, treatment poses a sizeable economic burden to patients. There are currently no conditions for coverage by the Quebec Public Prescription Drug Insurance Plan (elosulfase alfa can only be prescribed through exceptional patient status) and no formal guidelines that provide criteria for its use. Instead, there is considerable debate and policy discussion, particularly with regard to cost and which patients are most likely to benefit. Our data support the international and Canadian management guidelines for Morquio A, which recommend initiation of treatment as early as possible in light of the progressive nature of the disease [27, 28]. Given the clinical heterogeneity, individual treatment goals should be established by a multidisciplinary team taking into account the disease burden at the time of treatment initiation and the anticipated measurable benefits over time.

Several limitations of our study should be acknowledged. Though observational studies are useful in identifying relationships between characteristics, such as associations between genetic pathogenic variants and phenotype, causality cannot be established in the absence of a more stringent methodology involving required clinic visits and formal statistical analyses of variance with a complete data set. In addition, this study had no restrictions on surgery, other treatments, or activities before a clinic visit, and all could have influenced the assessments at that visit. 6MWT, respiratory function tests and echocardiograms were often performed locally, which may also have introduced some variability in the results. There were limitations with the scoring systems used and the expectation of improvement with treatment. For patients who have normal or near-normal scores in a test at baseline, stability may be an important outcome. Finally, our data are incomplete for several reasons: not all patients in Quebec with Morquio A syndrome gave consent for participation; many patients needed to travel long distances which may have resulted in missed data points; the 6MWT and MPS-HAQ were not routinely performed in all patients; assessments were not always performed in the recommended time window due to e.g. surgeries; and some patients were too young to complete the respiratory function tests. However, despite missing data, the available data were sufficient to show significant differences in clinical characteristics between the founder pathogenic variant and the non-founder pathogenic variant groups.

## Conclusions

Morquio A syndrome is a progressively debilitating lysosomal storage disorder that is heterogeneous in presentation and concentrated in the Quebecois population. We demonstrated a founder effect in French Canadians from Quebec with Morquio A, which manifests as a non-classical form of the disease related to the founder pathogenic variant in about half of the study population. Despite the observed founder effect, Quebecois patients did not appear to respond differently to therapy than patients in other Morquio A populations represented in clinical trials of elosulfase alfa. Improvements or stability in the 6MWT and/or MPS-HAQ in treated patients were seen regardless of *GALNS* pathogenic variant and were in line with those reported for patients in the clinical trials. Overall, the results of our study confirm published evidence for the beneficial effects of elosulfase alfa across multiple domains over both short and long time periods. In addition, they support the treatment plan proposed by the Canadian management guidelines, which involves initiation of treatment as early as possible, a cohesive follow-up approach, and establishment of individual treatment goals based on disease/functional stabilization and/or prevention of symptom onset [27]. Nevertheless, since enzyme replacement therapy with elosulfase alfa only partially improves the health and function of patients with Morquio A syndrome, continued research to develop additional disease-modifying treatments is needed.

## Data Availability

The datasets used and/or analysed during the current study are available from the corresponding author on reasonable request.

## Abbreviations

6MWT: 6-Min walk test
ADL: Activities of daily living
C6S: Chondroitin-6-sulfate
FEV_1_: Forced expiratory volume in 1 s
FVC: Forced vital capacity
GAGs: Glycosaminoglycans
GALNS: *N*-Acetylgalactosamine-6-sulfatase
ID: Patient identification number
KS: Keratan sulfate
MPS: Mucopolysaccharidosis
MPS-HAQ: Mucopolysaccharidosis Health Assessment Questionnaire
NA: Not available
NT: No elosulfase alfa treatment
